# Octa-Arginine Mediated Delivery of Wild-Type Lnk Protein Inhibits TPO-Induced M-MOK Megakaryoblastic Leukemic Cell Growth by Promoting Apoptosis

**DOI:** 10.1371/journal.pone.0023640

**Published:** 2011-08-10

**Authors:** Chung Yeng Looi, Miki Imanishi, Satoshi Takaki, Miki Sato, Natsuko Chiba, Yoji Sasahara, Shiroh Futaki, Shigeru Tsuchiya, Satoru Kumaki

**Affiliations:** 1 Department of Pediatrics, Tohoku University School of Medicine, Sendai, Japan; 2 Institute for Chemical Research, Kyoto University, Kyoto, Japan; 3 Research Institute, International Medical Center of Japan, Tokyo, Japan; 4 Department of Molecular Immunology, Institute of Development, Aging and Cancer, Tohoku University, Sendai, Japan; Heart Center Munich, Germany

## Abstract

**Background:**

Lnk plays a non-redundant role by negatively regulating cytokine signaling of TPO, SCF or EPO. Retroviral expression of Lnk has been shown to suppress hematopoietic leukemic cell proliferation indicating its therapeutic value in cancer therapy. However, retroviral gene delivery carries risks of insertional mutagenesis. To circumvent this undesired consequence, we fused a cell permeable peptide octa-arginine to Lnk and evaluated the efficacy of inhibition of leukemic cell proliferation in vitro.

**Methodology/Principal Findings:**

In this study, proliferation assays, flow cytometry, Western Blot analyses were performed on wild-type (WT), mutant Lnk R8 or BSA treated M-MOK cells. We found that delivered WT, but not mutant Lnk R8 blocked TPO-induced M-MOK megakaryoblastic leukemic cell proliferation. In contrast, WT Lnk R8 showed no growth inhibitive effect on non-hematopoietic HELA or COS-7 cell. Moreover, we demonstrated that TPO-induced M-MOK cell growth inhibition by WT Lnk R8 was dose-dependent. Penetrated WT Lnk R8 induced cell cycle arrest and apoptosis. Immunoprecipitation and Western blots data indicated WT Lnk R8 interacted with endogeneous Jak2 and downregulated Jak-Stat and MAPK phosphorylation level in M-MOK cells after TPO stimulation. Treatment with specific inhibitors (TG101348 and PD98059) indicated Jak-Stat and MAPK pathways were crucial for TPO-induced proliferation of M-MOK cells. Further analyses using TF-1 and HEL leukemic cell-lines showed that WT Lnk R8 inhibited Jak2-dependent cell proliferation. Using cord blood-derived CD34+ stem cells, we found that delivered WT Lnk R8 blocked TPO-induced megakaryopoiesis in vitro.

**Conclusions/Significance:**

Intracellular delivery of WT Lnk R8 fusion protein efficiently inhibited TPO-induced M-MOK leukemic cell growth by promoting apoptosis. WT Lnk R8 protein delivery may provide a safer and more practical approach to inhibit leukemic cell growth worthy of further development.

## Introduction

Cytokines play an important role in hematopoiesis. Myeloproliferative leukemia virus proto-oncogene (c-mpl) gene encodes a receptor for thrombopoietin (TPO) [1], a cytokine that potently stimulates megakaryocytopoiesis [2]. Ligand-induced c-mpl activation involves receptor homodimerization leading to recruitment and activation of Jak2 tyrosine kinase [3]. Jak2 is regulated by autoinhibitory JH2 domain and negative regulators such as suppressor of cytokine 3 (SOCS3) and Lnk [4], [5], [6]. Disruption of JH2 domain by constitutive active mutation of Jak2 V617F was discovered recently in human cancers and has a primary role in the pathogenesis of myeloproliferative neoplasms (MPN) [7], [8].

Lnk is an intracellular adaptor protein which expressed mainly in hematopoietic cells. Lnk plays a non-redundant role in negative regulation of several cytokine signalings by stem cell factor (SCF), TPO and erythropoietin (EPO) [6], [9], [10], [11]. Adaptor protein Lnk lacks kinase activities but possess interaction domains such as Proline-rich, Pleckstrin Homology (PH) and Src Homology 2 (SH2) domains. Lnk localizes to the plasma membrane via its PH domain and negatively regulates Jak2 tyrosine kinase through its SH2 domain [6], [10], [12], [13]. Lnk knockout mice showed marked expansion of hematopoietic precursors, B-lineage and megakaryocytes, whereas representation of these hematopoietic cells were reduced in Lnk transgenic mice, indicating important role of Lnk in hematopoiesis [10], [14], [15], [16], [17].

New therapeutic interventions have been made possible through the development of membrane-permeable carrier peptides [18]. Larger protein cargo have limitations to enter cells due to poor permeability of the cell plasma membrane but proteins conjugated with cell penetrating carrier peptides such as Transactivator of transcription (Tat) derived from Human Immunodeficiency Virus (HIV), Antennapedia (Antp) or polyarginine have been reported to transduce a wide range of therapeutically active proteins into living cells [19], [20], [21], [22]. Successful transduction using Antp peptide is limited in delivering small peptides or proteins [23]. Although Tat is more commonly used, recent studies showed that polyarginine exhibits even greater efficiency in terms of delivery of several peptides and proteins [24], [25], [26]. A detail study on polyarginine showed that the rate of cellular uptake was strongly correlated to the number of arginine residues present [25]. R8 (octamer) has been found to be optimum and demonstrated efficient internalization compared to R4 (tetramer) or R16 (hexadecamer) [25].

A model suggested that dysregulation of negative regulator such as Lnk may lead to development of cancer in human [12], [27]. Overexpression of Lnk using retroviruses has been shown to attenuate growth of hematopoietic leukemic cell-lines but not cells derived from solid tumors, indicating Lnk as a potent candidate in cancer therapy of hematopoietic-origin malignancies [28]. However, viral-based gene delivery often carries risk of insertional mutagenesis. Thus, we reasoned that expressing Lnk through protein delivery could be a safer approach in stopping leukemic cell growth. In this study, we generated full length wild-type (WT) Lnk protein fused to a membrane-permeable carrier peptide, octa-arginine (R8) and evaluated its efficacy in suppressing leukemic cell proliferation *in vitro*.

## Materials and Methods

### Ethics Statement

Umbilical cord blood was obtained from Miyagi Cord Blood Bank. All donors gave informed consent and the study was approved by Tohoku University Hospital Ethnic Committee.

### Cell culture

Human megakaryoblastic cell-line, M-MOK was maintained in RPMI medium with GM-CSF (20 ng/ml). Human erythroleukemic cell-line, TF-1 (ATCC: CRL-2003) was maintained in RPMI medium with IL-3 (10 ng/ml). HEL, human erythroleukemic cell-line with Jak2 V617F gain-of-function mutation (ATCC: TIB-180) was cultured in RPMI. HELA, human epithelial carcinoma cell-line (ATCC: CCL-2) and COS-7 fibroblast cell-line (ATCC: CRL-1651) were cultured in DMEM medium. All culture medium were supplemented with 10% Fetal Calf Serum (FCS), 100 units/ml penicillin, 100 µg/ml streptomycin and cells were grown in humidified 37°C with 5% CO_2_.

### CD34+ stem cells purification from cord blood

Mononuclear cells were isolated using Ficoll Hypaque (Pharmacia, Piscataway, NJ) density centrifugation. Cells were washed and resuspended in PBS with 2% FCS. Cells were incubated for 20 minutes at 4°C with biotin-conjugated anti-CD34 antibody (AbD Serotec, Oxford, UK). After washing, cells were incubated for 20 minutes at 4°C with anti-biotin microbeads (Miltenyi Biotech GmbH, Germany). Cells were washed twice and CD34+ stem cells were sorted with autoMACS cell separator (Miltenyi). More than 96% CD34+ stem cells were obtained in all preparations.

### Expression vectors

Human pcDNA3 Lnk cDNA was kindly provided by Dr. I. Nobuhisa (Kumamoto University, Japan). A mutant human Lnk (R392E mutation in SH2 domain) was generated by polymerase chain reaction (PCR)-based site-directed mutagenesis [13]. R8 and 6×His sequences were introduced into C terminal of human WT or mutant Lnk cDNA by PCR. The resulting pcDNA-WT or mutant Lnk-6×His-R8 construct was subcloned into NdeI and EcoRV sites of pET-29b bacterial expression vector (Novagen, Madison, WI). All constructs were verified by DNA sequencing.

### Protein expression and purification

WT or mutant Lnk R8 proteins were expressed in BL21 (DE3; Novagen). Cells were resuspended into extraction buffer (30 mM phosphate buffer, pH 7.8, 300 mM NaCl, 0.1% NP-40, 50 mM imidazole) with protease inhibitor cocktail (Roche Diagnostics, GmbH, Mannheim, Germany) before lysed by sonication for 1 minute in 4 cycles. Bacterial debris were pelleted, supernatant were subjected to affinity chromatography using His-bind affinity column (Qiagen, Hilden, Germany). Eluted protein was further purified with Heparin and ion exchange FPLC (Bio-Rad, Hercules, CA). Purified samples were desalted and buffers were exchanged into phosphate-buffered saline PBS (7.5) using PD-10 Sephadex G-25M column (GE healthcare, Piscataway, NJ) before application.

### Flow cytometry analysis of R8 fusion proteins uptake

To measure exogenous recombinant proteins uptake efficiency, M-MOK, TF-1, HEL, HELA or COS-7 cells were treated with 5 µM of fluorescein isothiocyanate (FITC)-labeled WT or mutant Lnk R8. After designated time, cells were collected, washed three times with PBS and trypsinized to remove membrane-bound proteins before subjected to flow cytometry analyses (BD Biosciences, San Jose, CA). Dead cells were gated out using Side Scatter and Propidium Iodide (PI) stain.

### Cell proliferation assays

2×10^4^ M-MOK cells were seeded in 24-well plates in 0.5 ml of RPMI before treated with designated concentration of WT, mutant Lnk R8 or BSA (Sigma, St Louis, MO) for 6 hours. Treated cells were supplemented with 10 ng/ml of TPO (Kirin Brewery Co., Tokyo, Japan) and further cultured. Viable cell count was performed every day.

1×10^4^ M-MOK, TF-1 or HEL cells were seeded in 96-well plates in RPMI prior to protein treatment. HELA or COS-7 cells were seeded at a density of 5×10^3^ cells in DMEM. Cells were treated with 5 µM of WT or mutant Lnk R8 or BSA. After 6 hours, 10% FCS (and cytokines TPO, GM-CSF or IL-3) were supplemented and samples were further cultured for 2 days. Cell viability was measured using Cell-Quanti MTT assays kit according to Manufacturer's instructions (BioAssay Systems, CA, USA). Absorbance was measured at 570 nm using Microplate Reader (Bio-Rad). All samples were done in triplicate. Cell viability was showed as ratio of absorbance (A_570nm_) in WT or mutant Lnk R8-treated cells relative to absorbance in BSA-treated cells.
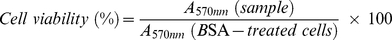



To study the effect of different inhibitors on TPO-induced M-MOK cell growth, 1×10^5^ cells per ml were seeded in 6-well plates in RPMI with 10% FCS. Cells were treated with 25 µM of TG101348, PD98059 or DMSO (inhibitor solvent) for 1 hour before TPO addition. Cell growth was determined by counting the number of viable cells for 3 days.

### Flow cytometry measurement of apoptosis

2×10^4^ M-MOK cells were treated with of WT, mutant Lnk R8 or BSA as described above. At designated time, M-MOK cells were harvested and stained with FITC- annexin V and PI (BD Biosciences) in binding buffer for 15 minutes. Stained cells were immediately subjected to flow cytometry analyses.

### Flow cytometry measurement of cell cycle

24 hours after TPO addition, protein-treated M-MOK cells were collected and washed three times with PBS .The supernatant was removed and 70% cold ethanol was added. After fixation, cells were treated with RNase for 1 hour in 37°C. Finally, cells were incubated in PI staining buffer and cell cycle was analyzed with flow cytometry. The percentages of cells in different cell cycle phases (G1, S, or G2 phase) were determined.

### Flow cytometry analysis of CD41+ megakaryocytes

1×10^5^ human CD34+ stem cells purified from cord blood were resuspended in X-VIVO 15 medium (BioWhittaker, Walkersville, MD) and treated with 5 µM of WT or mutant Lnk R8 or BSA. After 6 hours, X-VIVO 15 supplemented with 1% HSA (Human Serum Albumin) and TPO (100 ng/ml) were added to the medium. At day 0 and day 4, cells were collected, washed with PBS and labeled with mouse Phycoerythrin (PE)-CD41 antibody (Biolegend, San Diego, CA) or mouse PE-isotype control antibody (R&D Systems, Minneapolis, MN, USA). Cells were washed twice before subjected to flow cytometry analysis.

### Western blotting and immunoprecipitation

GM-CSF deprived M-MOK cells were treated with WT, mutant Lnk R8 or BSA prior to TPO stimulation. After designated time, all cells were harvested and lysed in lysis buffer (50 mM Tris, pH 8.0, 150 mM NaCl, 1% NP-40, 2 mM sodium orthovanadate, 100 mM NaF, 10 mM EDTA) containing protease inhibitors and 1.5 mM PMSF. The following antibodies were used: Lnk (Sigma; Abcam, Cambridge, UK), Jak2, pJak2 (Tyr1007/1008), Stat5, pMAPK (Thr202/Tyr204), cleaved PARP, β-actin, c-mpl (Santa Cruz Biotechnology, Santa Cruz, CA), MAPK, pStat5 (Y694) (Cell Signaling Technology, Beverly, MA, USA), pentaHis (Qiagen). For immunoprecipitation, lysates were precleared with protein G sepharose before adding anti-Jak2 or control IgG antibody (2 µg). After 2 hours rotation, the immunoprecipitates were collected by addition of protein G sepharose. Samples were resolved on 10% SDS-PAGE gel and blotted onto PVDF membrane (Milipore). Membranes were probed with above mentioned primary and corresponding secondary antibodies IgG TrueBlot conjugated to horseradish peroxidase (eBioscience, San Diego, CA). Membranes were developed using ECL enhanced chemiluminescence reagents (Amersham Biosciences).

### Fluorescent microscopy

WT or mutant Lnk R8 treated-M-MOK cells were stimulated with TPO. After 30 minutes, cells were collected, washed and briefly spun onto poly-L-lysine coated cover glass. Immunofluorescence staining was carried out according to previously described method with slight modification [29]. Cells were fixed with 4% paraformaldehyde for 20 minutes before permeabilized with PBS-T (0.1% Triton X-100) for 10 minutes. After 1 hour blocking in PBS-2% BSA, cells were stained with mouse anti-pentaHis (1∶100 dilution; Qiagen) and rabbit anti-Jak2 (clone C-20; 1∶100 dilution; Santa Cruz) antibodies for 1 hour. Cover glasses were washed three times with PBS and probed with Alexa Fluor-488 conjugated goat anti-mouse IgG (1∶500 dilution) and Alexa Fluor-594 conjugated goat anti-rabbit IgG (1∶500 dilution) secondary antibodies for 1 hour (Invitrogen). All antibodies incubation was performed in PBS-1% BSA at room temperature. After thorough rinsing, cover glasses were mounted on slides with ProLong Gold antiFade reagent (Invitrogen). Slides were observed using Olympus BX61 fluorescent microscope.

### Statistical analysis

Significance of differences between two groups was determined by Student's unpaired *t-test*. Statistical significance was presumed when *p<0.05, **p<0.01.

## Results

### Efficient uptake of WT or mutant Lnk R8 fusion proteins

**Figure 1:**
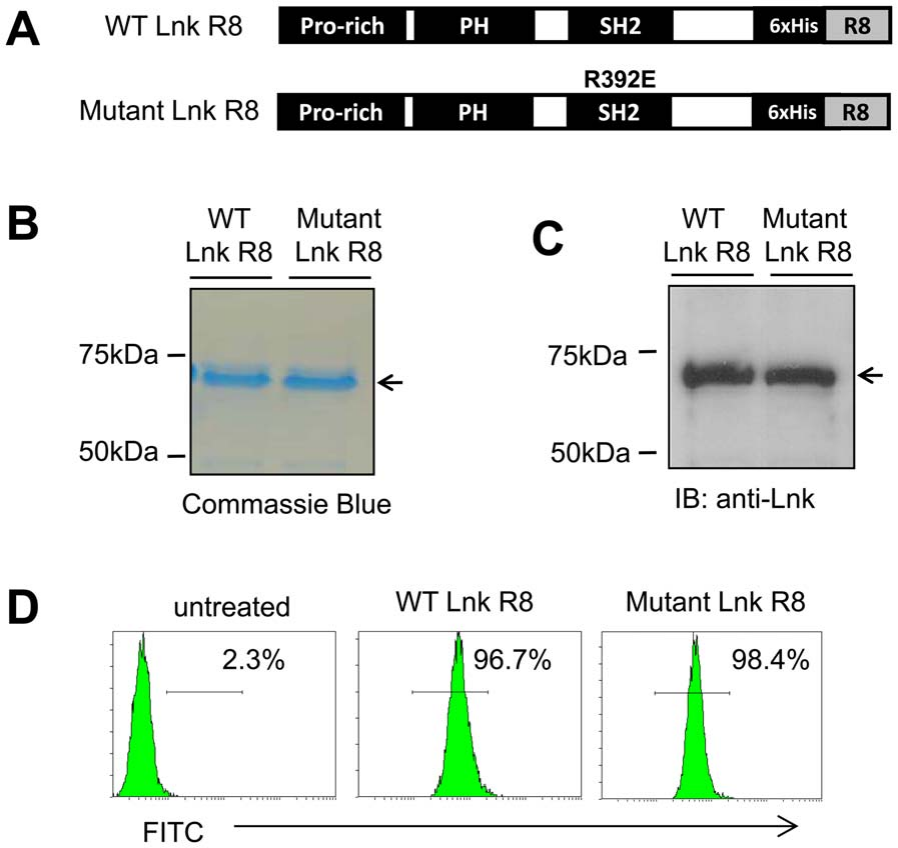
Efficient uptake of WT or mutant Lnk R8 fusion proteins. (A) Diagram of WT or mutant Lnk R8 constructs. Pro-rich:Proline-rich domain, PH:Pleckstrin homology domain, SH2:Src Homology 2 domain. (B) Coomassie blue staining of purified WT Lnk R8 or mutant Lnk R8. (C) Western blotting using anti-Lnk antibody confirmed the 70 kDa bands as WT and mutant Lnk R8. (D) Flow cytometry data showing internalization efficiency of FITC-labeled WT or mutant Lnk R8 in M-MOK cells.

To investigate the negative regulatory ability of WT Lnk, we included a Lnk mutant construct (R392E) which showed no inhibitive effect on TPO-induced cell growth in a previous report as control [13]. In order to promote cellular uptake of exogenous recombinant proteins, we fused a cell penetrating peptide octa-arginine (R8) to the C-terminal of WT or mutant Lnk and cloned these constructs into bacterial expression vectors (Figure 1A). Purified proteins were confirmed by Coomassie blue staining and Western blotting with anti-Lnk antibody (Figure 1B and 1C).

To determine the uptake efficiency of the R8 fusion proteins, M-MOK cells were co-cultured with 5 µM of fluorescein isothiocyanate (FITC)-labeled WT or mutant Lnk R8 at 37°C for 2 hours. Cells were harvested and trysinized before flow cytometry analyses. The uptake rate of WT or mutant Lnk R8 was 96.7% and 98.4% respectively (Figure 1D). Moreover, the uptake rates of WT or mutant Lnk R8 in different cell-lines ranged between 86∼97%, indicating efficient uptake of R8-fused proteins (Table 1).

**Table 1:** Uptake rate of Lnk R8 fusion proteins.

Cells	Uptake Efficiency (%)	
	WT Lnk R8	Mutant Lnk R8
TF-1	95.5	93.6
HEL	97.2	96.8
HELA	87.5	86.1
COS-7	94.2	94.9

Percentages of FITC-labeled WT or mutant Lnk R8 uptake efficiencies in TF-1, HEL, HELA or COS-7 cell-lines, as determined by flow cytometry analysis.

### WT Lnk R8 suppresses TPO-induced proliferation of M-MOK, but showed no inhibition effect on HELA or COS-7 cell growth

**Figure 2:**
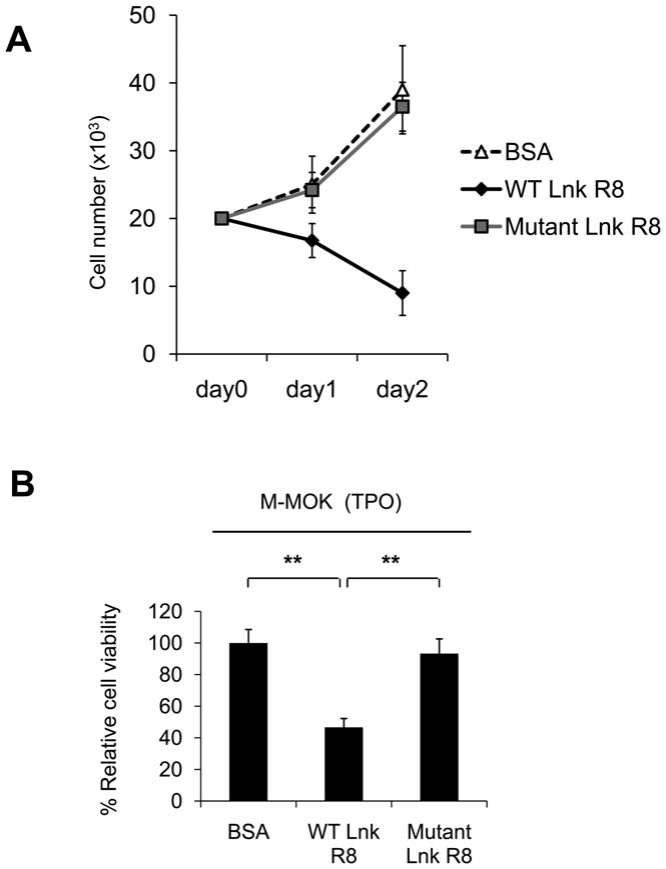
WT Lnk R8 inhibits TPO-induced proliferation of M-MOK. (A) M-MOK cells were pre-treated with the 5 µM of WT, mutant Lnk R8 or BSA. Viable cell numbers were determined at 24 and 48 hours after TPO addition. (B) M-MOK cells were treated as described above. Cell viability was measured by performing MTT assays at 48 hours after TPO addition. (Data represent mean ± SD and results were representative of three independent experiments, **p<0.01).

M-MOK is a megakaryoblastic cell-line established from acute megakaryoblastic leukemia patient in our laboratory [30]. M-MOK proliferates in response to TPO and it is a good model to study whether protein delivery of Lnk inhibits TPO-induced cell proliferation (Figure S1A and S1B) [31]. Cytokine deprived M-MOK cells were treated with 5 µM of WT, mutant Lnk R8 or BSA for 6 hours. TPO was added and cell count was performed at 24 and 48 hours time point. As shown in Figure 2A, WT Lnk R8 significantly inhibited TPO-induced cell growth. In contrast, mutant Lnk and BSA did not demonstrate any growth inhibition effect on M-MOK cells (*p<0.01*). To confirm the results of cell proliferation, we performed MTT assays to check cell viability after protein treatment. Cell viability of WT Lnk R8-treated cells was 46.6±5.6%, significantly lower compared to those of control groups, mutant Lnk R8 or BSA-treated cells, 93.3±9.3% and 100.0±8.5% respectively (Figure 2B).

**Figure 3:**
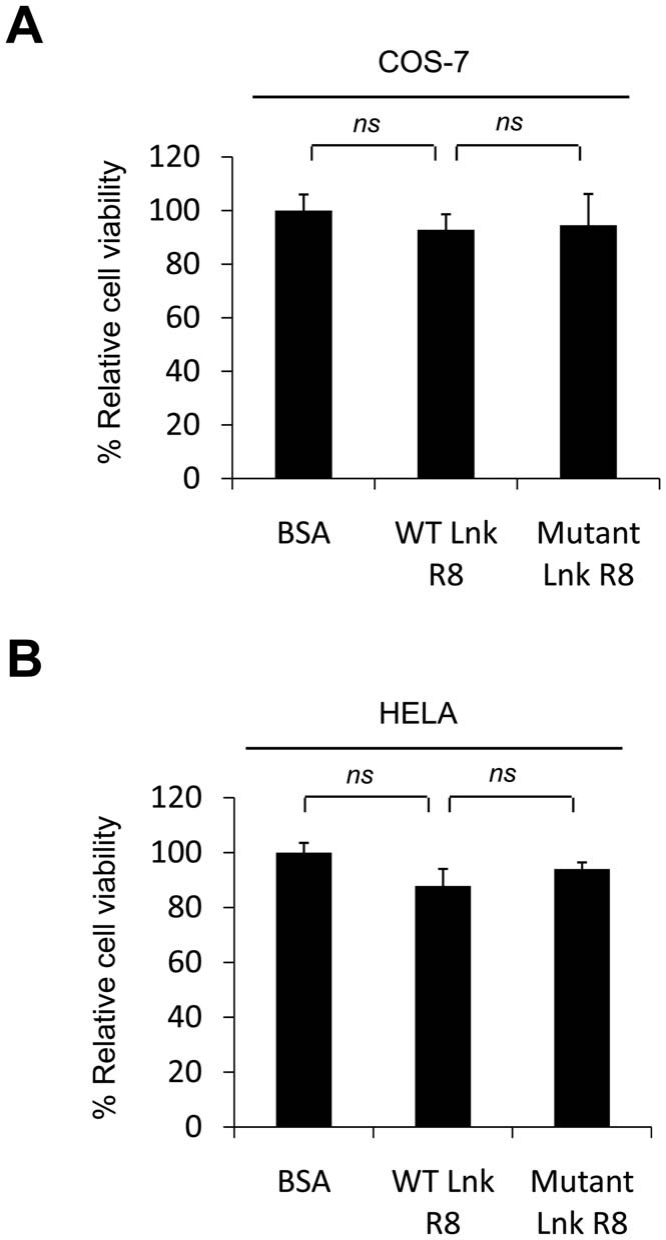
WT Lnk R8 delivery has no growth inhibition effect on non-hematopoietic COS-7 or HELA cells. (A) COS-7 or (B) HELA cell viability was measured by performing MTT assays at 48 hours after protein treatment. (*n.s.*: not significant).

To ensure that TPO-induced cell growth inhibition effect of WT Lnk R8 was not due to bacterial toxins contamination, we tested the R8 proteins in non-hematopoietic HELA or COS-7 cells. HELA or COS-7 cells were treated with WT, mutant Lnk R8 or BSA. After 48 hours, MTT assays were performed to check cell viability after protein treatment. Cell viability of WT Lnk R8-treated cells showed no significant difference compared to those of control groups, mutant Lnk R8 or BSA-treated cells (Figure 3A and 3B), thus excluding the possibility of bacterial toxins contamination.

### TPO-induced growth inhibition by WT Lnk R8 is dose-dependent

**Figure 4:**
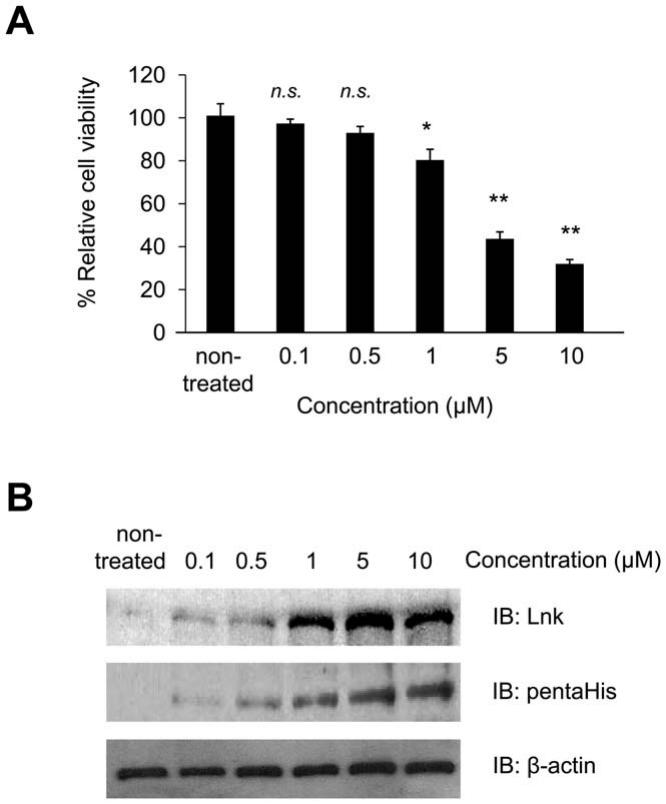
TPO-induced growth inhibition by WT Lnk R8 is dose-dependent. (A) Histogram showing percentages of M-MOK cell viability after treated with graded concentration of WT Lnk R8 relative to non-treated cells. Cell viability was determined at 48 hours after TPO stimulation through MTT assays. (*n.s.*: not significant; Data represent mean ± SD and results are representative of three independent experiments, *p<0.05, **p<0.01). (B) Western blotting data showing dose-dependent increment of delivered WT Lnk R8 proteins in M-MOK cells. Anti-Lnk antibody was used to probe both endogenous and intracellularly delivered Lnk proteins, whereas anti-pentaHis antibody probing showed delivered WT Lnk R8 proteins only. Anti-**β**-actin served as loading control.

Next, we examined the effect of graded WT Lnk R8 concentrations on TPO-induced M-MOK cell growth. Serum starved M-MOK cells were non-treated or treated with graded concentrations of WT Lnk R8 before TPO addition. Cell viability was determined at 48 hour time-point through MTT assays. Results showed that cell growth inhibition correlated with higher concentration of WT Lnk R8 proteins (Figure 4A). Moreover, we found that 1 µM of WT Lnk-R8 is the minimum concentration required to inhibit TPO-induced cell growth (Figure 4A). Western blotting with anti-pentaHis antibody confirmed a gradual increased of intracellular Lnk R8 (Figure 4B). In addition, anti-Lnk antibody showed that the amount of endogenous Lnk in M-MOK cells was very low compared to the delivered WT Lnk R8 protein (Figure 4B). Together, these data indicate that cell growth inhibition by WT Lnk R8 is dose-dependent.

### WT Lnk R8 induces cell-cycle arrest and promotes apoptosis

**Figure 5:**
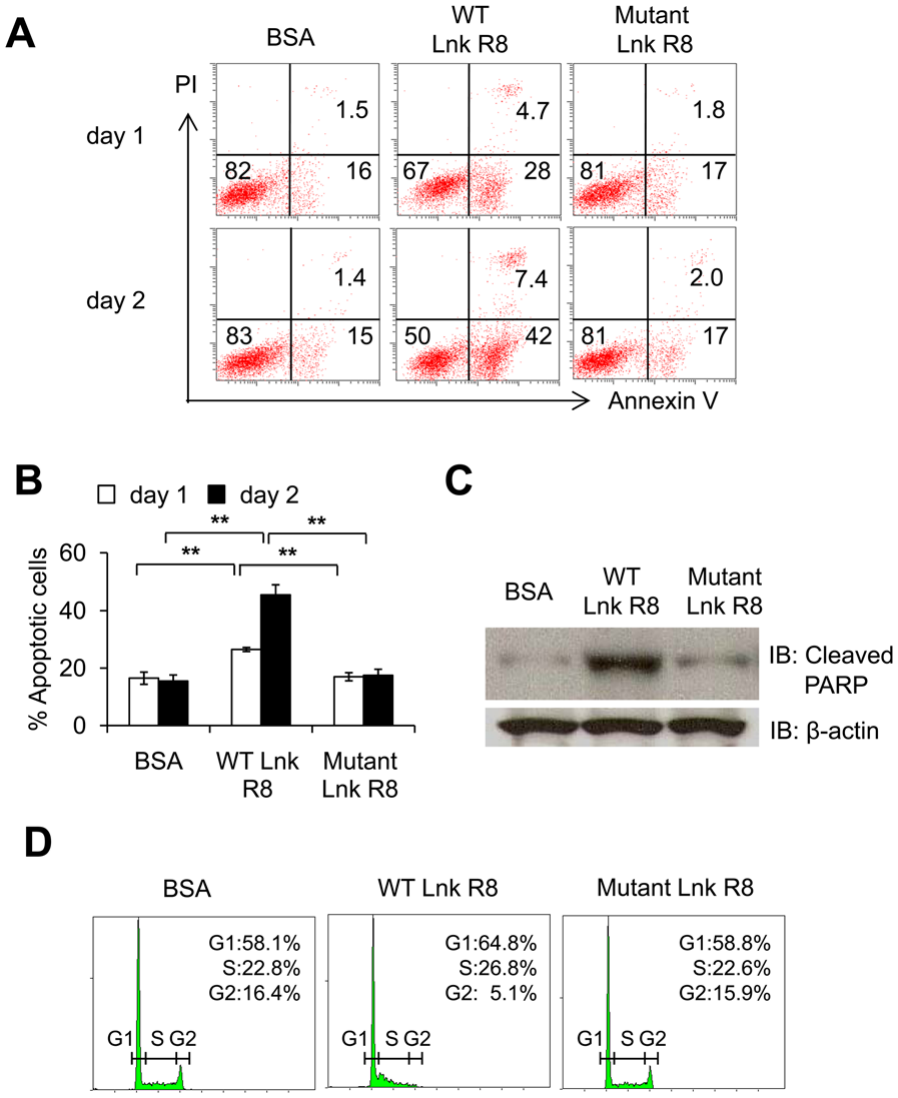
WT Lnk R8 induces cell cycle arrest and promotes apoptosis. (A) M-MOK cells were pre-treated with 5 µM of WT, mutant Lnk R8 or BSA for 6 hours before TPO addition. Cells were harvested at 24 or 48 hours after TPO stimulation. Cells were stained with FITC-Annexin V and PI to analyze apoptotic (Annexin V+ PI-) and dead (Annexin V+ PI+) cell fractions through flow cytometry. (B) Bar graph showing percentages of apoptotic cells for each treated sample at 24 or 48 hours after TPO addition. (Data represent mean ± SD and results are representative of three independent experiments, **p<0.01). (C) 48 hours after TPO addition, lysates from protein-treated M-MOK samples were subjected to Western blotting with cleaved PARP or **β**-actin antibodies. (D) 24 hours after TPO addition, protein-treated M-MOK samples were stained with PI and subjected to flow cytometry analysis. (Data represent one experiment of two independent experiments).

To find out the mechanism of TPO-induced cell growth inhibition, M-MOK cells were treated with WT, mutant Lnk R8 or BSA before adding TPO. After 24 and 48 hours, cells were stained with FITC-annexin V and propidium iodide (PI) to analyze percentage of apoptotic or dead cells through flow cytometry. On day one, the number of apoptotic cells in WT Lnk R8-treated M-MOK cells was 26.5±7.5%, significantly higher than those of control groups, mutant Lnk R8 or BSA, 17±1.4% or 16.5±2.1% respectively (Figure 5A and 5B). On day two, the number of apoptotic cells in WT Lnk R8-treated cells increased to 45.5±3.5%, two-fold higher compared to mutant Lnk R8 or BSA (Figure 5A and 5B). Next, we collected lysates of protein treated-M-MOK cells after 48 hours and performed Western blotting using cleaved PARP antibody, another marker for cells undergoing apoptosis. Again, result showed higher amount of cleaved PARP in WT Lnk R8-treated cells compared to mutant Lnk or BSA-treated cells (Figure 5C).

To further elucidate the mechanism of TPO-induced cell growth inhibition, we investigated the effect of WT Lnk R8 on cell cycle distribution 24 hours after TPO addition. WT Lnk R8-treated cell population in the G2 phase was three-fold lower compared to mutant Lnk R8 or BSA–treated cells (Figure 5D). In contrast, G1 and S phase population were higher in WT Lnk R8-treated cells (65% and 27%, respectively) compared to mutant Lnk or BSA group, indicating WT Lnk R8 blocked S-phase progression of M-MOK cells after TPO stimulation (Figure 5D).

### WT Lnk R8 binds endogenous Jak2 and impairs TPO-mediated Jak-Stat and MAPK activation

**Figure 6:**
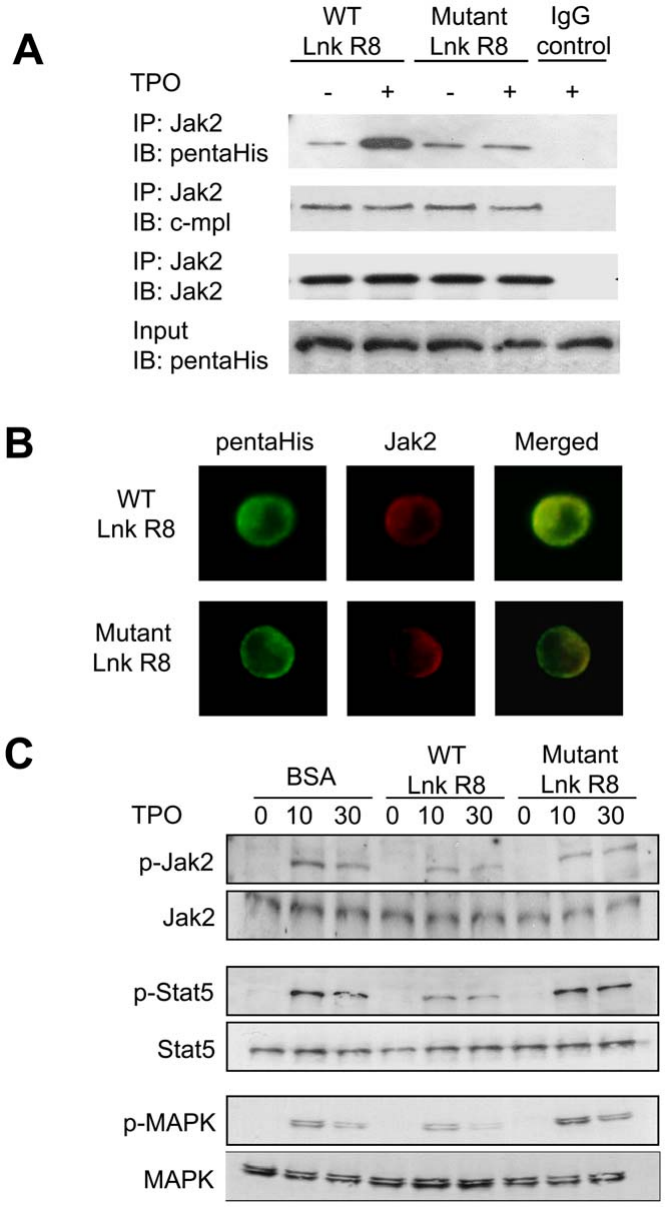
WT Lnk R8 binds endogenous Jak2 and impairs TPO-mediated Jak-Stat and MAPK activation. (A) M-MOK cells treated with WT or mutant Lnk R8 were either stimulated or not stimulated with TPO. Cells were collected and thoroughly washed before lysed. Lysates were immunoprecipitated with anti-Jak2 or control IgG antibody and analyzed by Western blotting with anti-Jak2, anti-pentaHis or anti-c-mpl antibodies. IgG control lane represents immunoprecipitation from WT Lnk R8-treated sample (with TPO stimulation) using control IgG antibody. (B) Protein-treated M-MOK cells were stimulated with TPO for 30 minutes. Cells were fixed and probed with anti-pentaHis and anti-Jak2 antibodies. Secondary Alexa 488 and Alexa 594 conjugated antibodies were used to detect primary antibodies (Green: Lnk R8, Red: Jak2). Co-localization of Lnk R8 and Jak2 (yellow) were shown in merged pictures. (C) Protein treated-M-MOK cells were stimulated with 10 ng/ml of TPO for 0, 10, 30 minutes. Cells were harvested and lysates were subjected to Western blot analyses. Phosphorylation and total protein levels of Jak2, Stat5 and MAPK were detected using antibodies as described in Materials and Methods.

To investigate whether penetrated WT Lnk R8 interacted with Jak2, we did immunoprecipitation with Jak2 or control IgG antibodies using lysates from Lnk R8-treated M-MOK cells stimulated with or without TPO. WT, but not mutant Lnk R8 strongly bound with Jak2 after TPO stimulation (Figure 6A). Immunofluorescence study showed WT Lnk R8 co-localized with Jak2 after TPO stimulation, whereas mutant Lnk R8 partially co-localized with Jak2 probably due to a weak interaction (Figure 6B). Next, we examined downstream signals activated by c-mpl (TPO receptor) 0, 10 or 30 minutes after TPO addition in WT, mutant Lnk R8 or BSA-treated M-MOK cells. Phosphorylation level of Jak2, Stat5 or MAPK was significantly downregulated in WT Lnk R8-treated cells compared to mutant Lnk R8 or BSA-treated cells (Figure 6C). Whereas, total Jak2, Stat5 and MAPK protein levels were not affected indicating lower phosphorylation level was not due to reduction in protein loading.

### TG101348 and PD98059 blocks TPO-induced proliferation of M-MOK cells

**Figure 7:**
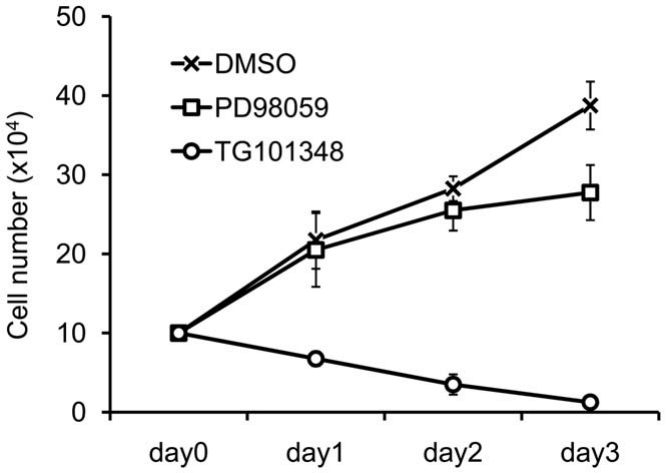
TG101348 and PD98059 suppress TPO-induced growth of M-MOK cells. M-MOK cells were pre-treated with either 25 µM of Jak2 inhibitor (TG101348), MAPK inhibitor (PD98059) or solvent DMSO for 1 hour before TPO addition. Cell proliferation was determined by counting viable cell numbers for 3 consecutive days. (Data represent mean ± SD and results are representative of three independent experiments).

To determine whether these pathways involved in TPO-induced cell growth, M-MOK cells were pretreated with recently developed specific Jak2 inhibitor, TG101348 [32], PD98059 (MAPK inhibitor) or inhibitor solvent DMSO for 1 hour in RPMI with 10% FCS before TPO addition. Each day cell count was performed to check cell number. DMSO has no effect on TPO-induced cell proliferation (Figure 7). In contrast, TG101348 significantly attenuated the proliferation of M-MOK cells (Figure 7). PD98059 only partially inhibited the growth of M-MOK cells, probably due to the presence of other survival signals such as Jak-Stat (Figure 7). Together, these data show that Jak-Stat and MAPK signaling molecules are crucial for TPO-induced M-MOK cell proliferation.

### WT Lnk R8 suppresses Jak2-dependent proliferation of leukemic cell-lines

**Figure 8:**
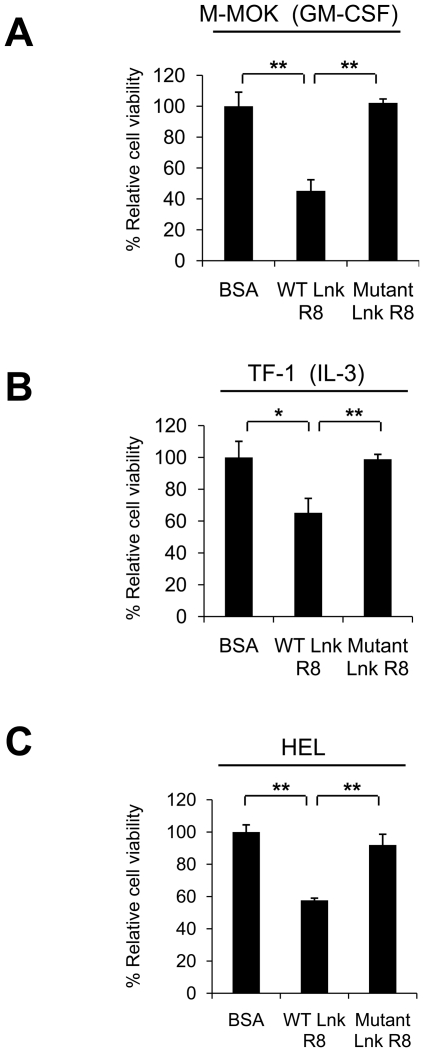
WT Lnk R8 inhibits Jak2-dependent cell proliferation. (A) M-MOK, (B) TF-1 or (C) HEL cells were pre-treated with the 5 µM of WT, mutant Lnk R8 or BSA. After treatment, cytokines GM-CSF and IL-3 were added into culture medium of M-MOK and TF-1, respectively. Cell viability was measured by performing MTT assays after 2 days. (Data represent mean ± SD and results are representative of three independent experiments, *p<0.05, **p<0.01).

Next, we would like to know whether WT Lnk R8 is effective in other Jak2-dependent signaling such as GM-CSF or IL-3. M-MOK cells also proliferate in response to GM-CSF [30], whereas TF-1 cells grow in response to IL-3 [33]. Cytokine-deprived M-MOK or TF-1 cells were treated with WT, mutant Lnk R8 or BSA. Cytokines (GM-CSF or IL-3) were added and MTT assays were performed after 48 hours. WT Lnk R8 significantly inhibited GM-CSF or IL-3-induced cell growth compared to those of control groups, mutant Lnk R8 or BSA-treated cells (Figure 8A and 8B). Another Jak2-dependent leukemic cell-line, HEL proliferate in the absence of cytokine such as EPO due to Jak2 V617F gain-of-function mutation [34]. As shown in Figure 8C, we found that WT Lnk R8 effectively attenuated Jak2 V617-induced growth of HEL cells.

### WT Lnk R8 inhibits TPO-induced megakaryopoiesis *in vitro*

**Figure 9:**
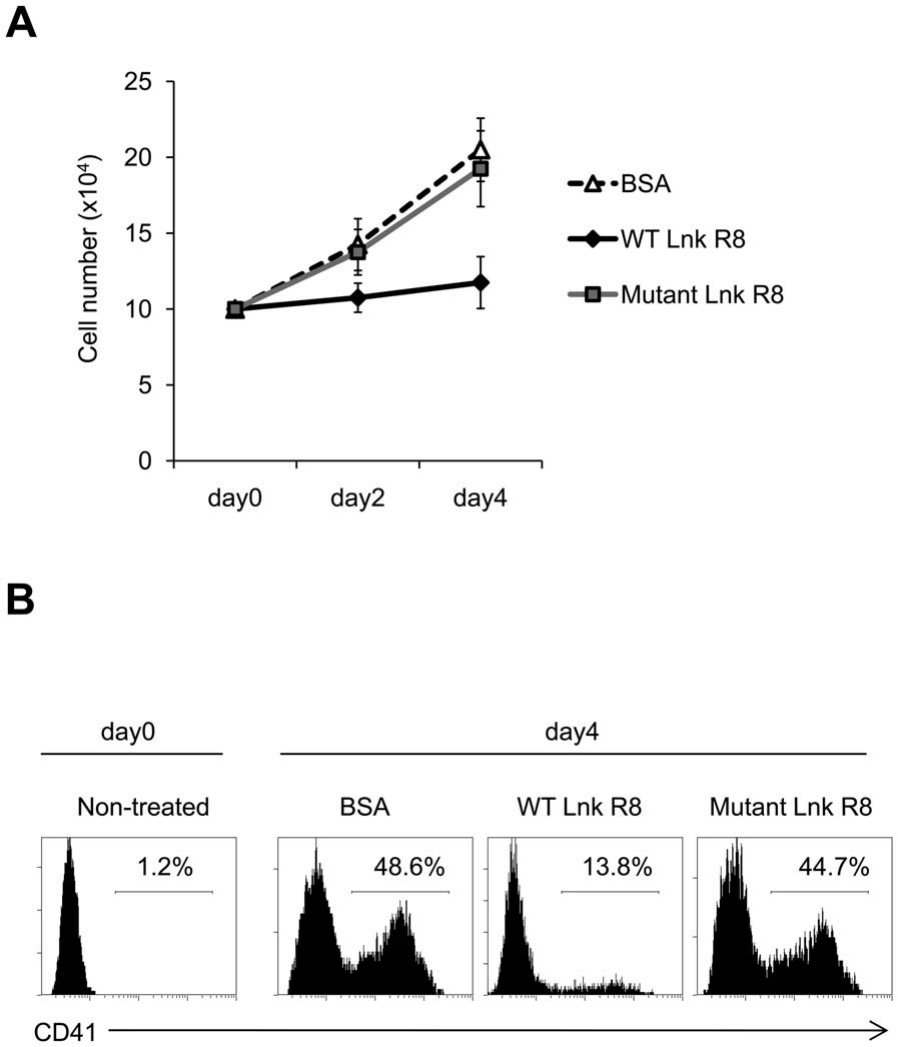
WT, but not mutant Lnk R8 blocks TPO-induced megakaryopoiesis. (A) CD34+ cells were treated with WT, mutant Lnk R8 or BSA as described in Materials and Methods and cultured for 4 days in the presence of TPO. Viable cell numbers were determined by trypan blue exclusion every two days. (B) BSA or Lnk R8 proteins treated-CD34+ cells were subjected to flow cytometry analysis of CD41+ megakaryocytes at day 0 and day 4 after TPO addition. (Data represent one experiment of two independent experiments).

Previous report showed that overexpression of WT Lnk blocked murine megakaryopoiesis [10]. To examine whether WT Lnk R8 could block megakaryocytosis, CD34+ stem cells were purified from donor cord blood. The uptake rates of Lnk R8 proteins in CD34+ cells were >90% (data not shown). Next, we treated CD34+ stem cells with Lnk R8 or BSA proteins before TPO addition. As shown in Figure 9A, WT Lnk R8, but not mutant Lnk or BSA blocked TPO-induced cellular expansion. 4 days after TPO stimulation, the percentages of CD41+ megakaryocytes increased to 48.6% and 44.7% in BSA and mutant Lnk R8-treated cells, respectively (Figure 9B). In contrast, the percentage of CD41+ megakaryocytes was significantly lower in WT Lnk R8-treated samples (13.8%), indicating delivered WT Lnk R8 blocked TPO-induced megakaryopoiesis *in vitro* (Figure 9B).

## Discussion

Retroviral overexpression of Lnk strongly attenuated the growth of hematopoietic leukemic cell-lines but not cells derived from solid tumors, indicated potential therapeutic value of Lnk in cancer therapy [28]. Viral-based gene delivery could result in genomic modification of human cells which carries risk of insertional mutagenesis [35]. In 2006, we introduced the idea of co-expressing ganciclovir-inducible HSVtk (Herpes Simplex Virus thymidine kinase) suicide gene to offset the adverse effect by viral vector if leukemogenesis occurs [36]. Throughout the years, safer lentiviral vector has been developed for gene therapy, but practical use in human still remained controversial [37]. On the other hand, the recent successes and relatively lower genomic modification risk by protein transduction through fusion with a cell penetrating peptide prompted us to evaluate the effectiveness of Lnk protein in stopping leukemia cell growth *in vitro*.

In this study, we showed that R8 was sufficient to induce efficient uptake of fusion proteins into cells. Reducing the number of arginine residues from R11 to R9 has been reported to improved solubility of proteins [38], but further reduction of arginine residues (R4 or R6) resulted in reduced uptake efficiency [25]. Previous studies showed that the intake of polyarginine-fused proteins were concentration and time-dependent [25], [38]. We showed that concentration below 1 µM was insufficient to inhibit TPO-induced M-MOK cell growth. Lower concentration led to fewer uptakes of WT Lnk R8 protein and less efficient cell growth inhibition. Using graded concentrations of WT Lnk R8, we estimated that half maximal inhibitory concentration (IC50) of WT Lnk R8 was ∼5 µM.

The exact protein transduction mechanism by octa-arginine is not known but Nakase et al. suggested that octa-arginine peptides are internalized through macropinocytosis [39]. However, some cells showed punctuate staining at the plasma membrane which did not diminish after several times of washing with PBS. Due to this reason, trypsinization treatment prior to flow cytometry analysis is required to eliminate membrane-bound R8 fusion proteins. A possible explanation on why octa-arginine showed strong adsorption to the cell surface may be due to their high basic charges [40]. Evidences suggested that high basic charges of arginine-rich peptides, such as Tat or polyarginine, may facilitate interaction with negative charges of plasma membrane which then induced internalization of these peptides into the cells [40], [41], [42].

In this study, we showed that WT Lnk R8 delivery abrogated TPO-induced cell growth and induce apoptosis in M-MOK cells. Apart from TPO signaling, WT Lnk R8 is also an effective growth inhibitor in other Jak2-mediated cytokine signaling such as GM-CSF and IL-3. In contrast, WT Lnk R8 showed no growth inhibition effect on HELA and COS-7 cell growth, indicating WT Lnk R8 has no effect on non-hematopoietic cells. On the other hand, CD34+ stem cells treated with WT Lnk R8 resulted in lower cellular expansion and CD41+ megakaryocytes formation, indicating TPO activation is also important in providing proliferation and differentiation signals in primary human CD34+ stem cells. As WT Lnk R8 may have a general effect on cytokine-dependent hematopoiesis (TPO-induced megakaryopoiesis) not restricted to leukemic cell growth, thus, it should be cautioned that WT Lnk R8 treatment could carry risk such as thrombocytopenia although further studies are needed to elucidate this.

Lnk has been shown to interact with major binding site phosphorylated (pY813) and minor pY613 in Jak2 through its SH2 domain after TPO stimulation [27]. Through immunoprecipitation and Western blot analysis, we showed that WT, but not mutant Lnk R8, bound strongly to endogenous Jak2 in the presence of TPO. The presence of relatively weak interaction between Lnk and Jak2 before TPO stimulation can be explained by a recent study, which revealed a novel moderate interaction involving the N-terminal region of Lnk [43]. We also detected the presence of c-mpl in WT Lnk immunoprecipitates, indicating Lnk formed a complex with Jak2/c-mpl and attenuated phosphorylation level of Jak-Stat and MAPK after TPO stimulation. Using specific inhibitors, we demonstrated that both Jak-Stat and MAPK were crucial for TPO-induced cell growth. MAPK and STAT5 have been implicated in the regulation of genes that are important for the prevention of apoptosis, proliferation, cell cycle progression in hematopoietic cells [44], [45]. Thus, blocking these surviving signals could switch the balance from cell growth to cell death, resulting in increased rate of apoptosis as observed in WT Lnk R8-treated M-MOK cells.

Recently, Lnk mutations have been discovered in two Jak2 V617F-negative MPN patients resulting in aberrant Jak-Stat activation due to loss of Lnk negative feedback regulation [46]. Further analysis performed in 61 patients showed that Lnk mutations targeting an exon 2 ‘hot spot’ in the PH domain were prevalent in blast-phase post-primary myelofibrosis (PMF) [47]. Intriguingly, another Jak2 negative regulator, SOCS2 has been found to be epigenetically downregulated in two out of seven MPN patients, suggesting lack of negative regulator might be an important second step in the genesis of cytokine-independent MPN clones [48]. In this report, we showed that intracellularly delivered WT Lnk R8 retained the ability to negatively regulate Jak2 tyrosine kinase after TPO stimulation. Thus restoring Lnk function through protein delivery could be a safer and potential way in treating MPN patient with loss-of-function Lnk mutation.

In this study, we have demonstrated the efficiency and inhibition mechanism of TPO activation by WT Lnk R8 in M-MOK leukemic cells. In addition, WT Lnk R8 is also effective against other Jak2-dependent cytokine cell growth. Based on our *in vitro* data shown here, the potential of WT Lnk R8 can be further investigated in MPN animal model to evaluate its efficacy as a method of cancer therapy. Moreover, the function of WT Lnk R8 can be enhanced by fusing with Hemagglutinin-2 subunit peptides (HA2), where previous study showed that HA2 fusion proteins remarkably reduced protein entrapment in the micropinosomes and enhanced the function of delivered protein at a lower dose [38].

## Supporting Information

Figure S1 **(A) GM-CSF deprived M-MOK cells were cultured with or without TPO.** Cells were counted for 3 consecutive days. (Data represent mean ± SD and results are representative of three independent experiments). (B) Lysates from unstimulated or TPO-stimulated M-MOK cells were immunoprecipitated with c-mpl antibody. Western blots showing immunoprecipitated c-mpl and total tyrosine phosphorylation detected with c-mpl and 4G10 anti-tyrosine phosphorylation antibodies, respectively.
(PDF)
