# Supplementary figures and images for: Octa-Arginine Mediated Delivery of Wild-Type Lnk Protein Inhibits TPO-Induced M-MOK Megakaryoblastic Leukemic Cell Growth by Promoting Apoptosis
Source: PLoS One. 2011 Aug 10;6(8):e23640. doi: 10.1371/journal.pone.0023640 (PMC3154509; doi:10.1371/journal.pone.0023640)

Figure S1

A

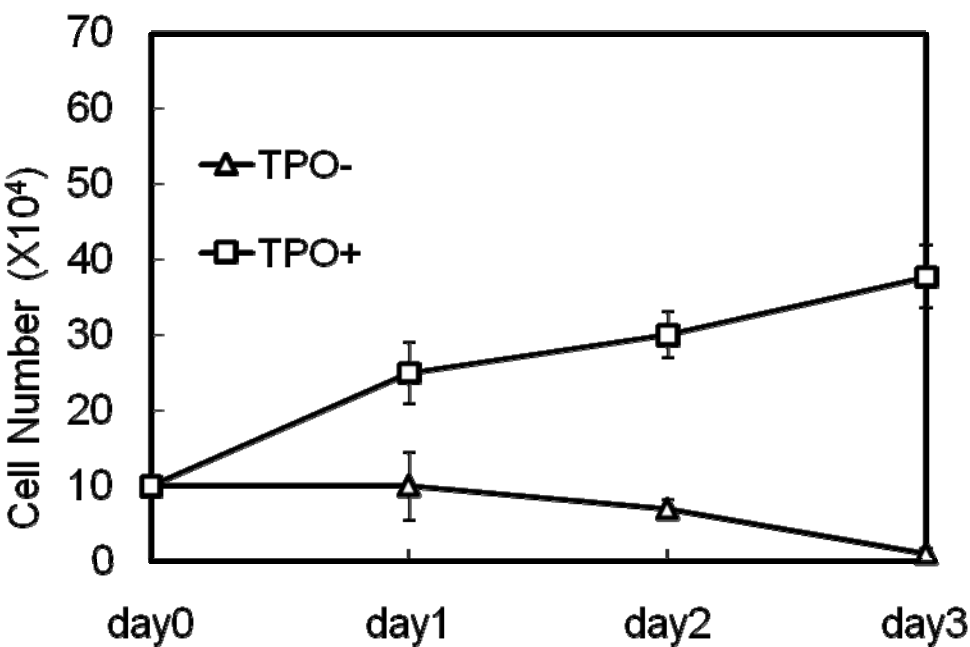

B

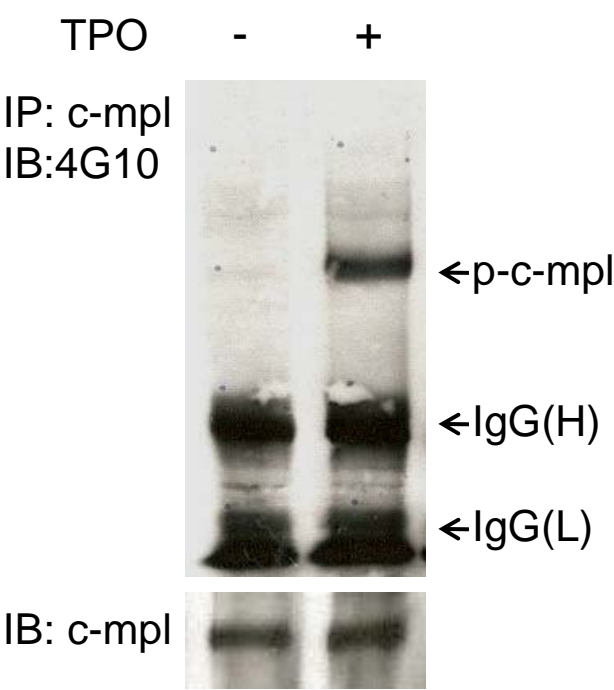

Supplement: Figure S1 — (A) GM-CSF deprived M-MOK cells were cultured with or without TPO. Cells were counted for 3 consecutive days. (Data represent mean ± SD and results are representative of three independent experiments). (B) Lysates from unstimulated or TPO-stimulated M-MOK cells were immunoprecipitated with c-mpl antibody. Western blots showing immunoprecipitated c-mpl and total tyrosine phosphorylation detected with c-mpl and 4G10 anti-tyrosine phosphorylation antibodies, respectively. (PDF) [file pone.0023640.s001.pdf]
